# Comparative Effects of Experimental Bioactive Glasses on Dentin Permeability and Tubule Occlusion In Vitro

**DOI:** 10.3390/jfb17060302

**Published:** 2026-06-18

**Authors:** Julia Moro Destro, Bruna de Oliveira Reis, Daniela Alvim Chrisostomo, Henrico Badaoui Strazzi-Sahyon, Mariana Elias Queiroz, Francine Benetti, Ticiane Cestari Fagundes, Marina Trevelin Sousa, Edgar Dutra Zanotto, Luciano Tavares Angelo Cintra, Andre Luiz Fraga Briso, Paulo Henrique dos Santos

**Affiliations:** 1 Department of Dental Materials and Prosthodontics, Aracatuba School of Dentistry, São Paulo State University, Aracatuba 16015-050, SP, Brazil; juliamorod@gmail.com (J.M.D.); mariana.equeiroz@gmail.com (M.E.Q.); 2 Department of Restorative Dentistry, Focus Educational Group, Teresina 64052-280, PI, Brazil; bruna_dol@hotmail.com; 3 Faculty of Dentistry, University of Toronto, Toronto, ON M5G 1X3, Canada; daniela.chrisostomo@mail.utoronto.ca; 4 Department of Restorative Dentistry and Endodontics, Aracatuba School of Dentistry, São Paulo State University, Aracatuba 16015-050, SP, Brazil; ico_strazzi@hotmail.com (H.B.S.-S.); ticiane.fagundes@unesp.br (T.C.F.); luciano.cintra@unesp.br (L.T.A.C.); andre.briso@unesp.br (A.L.F.B.); 5 Department of Restorative Dentistry, School of Dentistry, Federal University of Minas Gerais (UFMG), Belo Horizonte 31270-901, MG, Brazil; francine-benetti@ufmg.br; 6 Vitreous Materials Laboratory (LaMaV), Department of Materials Engineering, Federal University of Sao Carlos (UFSCar), Sao Carlos 13565-905, SP, Brazil; marina.trevelin@gmail.com (M.T.S.); dedz@ufscar.br (E.D.Z.)

**Keywords:** dentin sensitivity, dentin desensitizing agents, dentin permeability

## Abstract

This study aimed to evaluate the effects of experimental bioactive glass solutions on dentinal fluid flow and hydraulic conductance in vitro. Dentin blocks of 50 bovine incisors were divided into 5 groups according to the desensitizing agent used (*n* = 10): Group 1—control (no treatment); Group 2—Bioglass^®^ 45S5; Group 3—Bioglass^®^ F18; Group 4—Biosilicate gel; Group 5—Desensibilize Nano P (FGM). Treatments were applied for 20 min daily over 15 days. The specimens were subjected to a citric acid challenge to simulate oral demineralizing conditions. Dentinal fluid flow and hydraulic conductance were evaluated before and after the desensitizing procedures and the acidic challenge. Scanning electron microscopy (SEM) provided qualitative dentin surface analysis. Dentinal fluid flow and hydraulic conductance data were analyzed by two-way repeated measures ANOVA and Tukey’s test (*α* = 0.05). The control group showed the highest dentinal fluid flow and hydraulic conductance values after the acid challenge (*p* < 0.05). After product application, Bioglass^®^ 45S5 and Bioglass^®^ F18 showed the lowest dentinal fluid flow (0.463 ± 0.124 Lp and 0.549 ± 0.239 Lp, respectively) and hydraulic conductance values (0.025 ± 0.007 Lp and 0.026 ± 0.007 Lp, respectively), differing significantly from Biosilicate Gel and Desensibilize Nano P (*p* < 0.0001). Biosilicate Gel and Desensibilize Nano P showed higher dentinal fluid flow (0.864 ± 0.180 Lp and 0.840 ± 0.173 Lp, respectively) and hydraulic conductance values (0.047 ± 0.010 Lp and 0.046 ± 0.009 Lp, respectively) after application (*p* < 0.0001). After the acid challenge, no significant differences were observed among the groups (*p* > 0.05), although all experimental groups showed numerically lower values than the control group. Bioactive glass-based desensitizing agents reduced dentinal fluid flow and hydraulic conductance, suggesting dentinal tubule occlusion. Bioglass^®^ 45S5 and Bioglass^®^ F18 showed the most stable performance, maintaining lower values even after the acid challenge, suggesting potential for the management of dentin hypersensitivity.

## 1. Introduction

Dentin hypersensitivity is a clinical condition characterized by sharp, short-lasting pain resulting from dentin exposure in response to various oral stimuli, including thermal changes (e.g., cold temperatures), airflow, evaporative, tactile, osmotic, or chemical stimuli [1,2]. It is commonly associated with enamel loss due to abrasion, abfraction, erosion, gingival recession, or cementum loss following periodontal treatment [3,4]. The most widely accepted mechanism underlying dentin hypersensitivity was proposed by Brännström [1,5]. According to this theory, pain-inducing stimuli either increase dentinal fluid flow or change its direction, thereby activating nerves surrounding the odontoblasts and resulting in pain sensation [1]. To manage this condition, desensitizing agents are commonly applied. These agents may act through two main mechanisms: reducing the excitability of intradental nerve endings and occluding dentinal tubules [6,7]. In line with the hydrodynamic theory, therapeutic materials that reduce dentinal fluid movement may help alleviate dentin hypersensitivity, particularly by decreasing dentinal tubule permeability through occlusion or mineral deposition [8].

Despite the numerous physicochemical treatment agents available for dentin hypersensitivity, none is currently considered ideal [9]. In addition, there is no gold standard method, and adequate in vivo evidence for the treatment of dentin hypersensitivity remains limited [2]. Several treatment approaches have been proposed, including mouthwashes and toothpastes containing desensitizing chemical agents such as potassium nitrate, aluminum ferric oxalate, carbonate, and fluoride compounds [9]. Similarly, physical approaches, including fluoride varnishes, bonding agents, and restorative treatments such as composite resins, have also been used [10]. Additional desensitizing agents reported in the literature include calcium-based compounds, oxalates, strontium chloride, fluorides [9], high- and low-intensity lasers [3,9,11], and glutaraldehyde [12]. The mechanism of action of most treatment methods involves either reducing the neural response to stimuli, as observed with potassium salts (e.g., potassium nitrate), or promoting dentinal tubule occlusion, as occurs with fluoride and oxalate compounds. This occlusion may reduce fluid movement within dentin and, consequently, help reduce pain [13,14]. Some commercial products, such as Desensibilize Nano P, which contain nanometric calcium phosphate, sodium fluoride, and potassium nitrate, have been proposed to provide a dual mechanism of action by combining dentinal tubule occlusion with biomimetic remineralization. However, some of these treatments are considered temporary. Therefore, an ideal treatment for dentin hypersensitivity would ideally provide long-term effectiveness while remaining affordable and easy to use [13].

More recently, bioactive glasses have received increasing attention for their role in dentin hypersensitivity management [1,15,16,17,18,19,20]. Compared with conventional treatments, these materials promote biomimetic remineralization by forming a stable hydroxyapatite-like layer on the dentin surface, promoting dentinal tubule occlusion and potentially reducing hypersensitivity, with clinical performance comparable or superior to traditional agents, particularly in the short term [19,21]. Among bioactive materials, bioactive glass and hydroxyapatite are considered primary candidates due to their ability to simultaneously induce remineralization and occlude exposed dentinal tubules [22,23,24,25]. Originally developed for bone regeneration, bioactive glass has been increasingly adapted for dental tissue repair applications [26,27]. Its biological activity has demonstrated promising therapeutic outcomes, including pulp regeneration [28,29], remineralization of dental hard tissues [30,31], and potential use as a scaffold material [32]. The effectiveness of bioactive glass in reducing dentin hypersensitivity has been primarily attributed to its capacity to induce dentin remineralization and tubule occlusion [33], while its ability to promote calcium phosphate precipitation in physiological environments may contribute to its application in remineralization and hard tissue regeneration [1,34,35]. Furthermore, its remineralization potential, combined with an appropriate particle size, enhances tubule occlusion, supports mineral deposition, and inhibits dentin demineralization [1,36,37], while its antimicrobial activity may contribute to reducing bacteria-induced pulpal responses associated with hypersensitivity [1,38].

However, despite significant advancements in materials for the treatment of dentin hypersensitivity, this condition continues to affect a large proportion of the population and remains challenging to manage. Furthermore, the long-term effectiveness of these materials is often limited, as their performance may be compromised by variations in the oral environment, including fluctuations in pH and other physicochemical conditions. Therefore, the findings of this study may provide a basis for future investigations exploring the potential application of bioactive glass-based desensitizing agents in the management of dentin hypersensitivity. The objective of the present study was to evaluate the effects of different desensitizing agents on dentin subjected to acid challenge by assessing dentinal fluid flow and hydraulic conductance in vitro. The null hypotheses tested were as follows: (1) There would be no change in dentinal fluid flow and hydraulic conductance after treatment with different desensitizing agents, and (2) there would be no change in dentinal fluid flow and hydraulic conductance of specimens treated with different desensitizing agents after acid challenge.

## 2. Materials and Methods

### 2.1. Experimental Design

In this study, the response variables were dentinal fluid flow and hydraulic conductance values (both acting complementarily but assessed using the same test with the same purpose). The study analyzed two factors: (1) the type of desensitizing agent at five levels (control—no product applied, Bioglass^®^ 45S5, Bioglass^®^ F18, Biosilicate gel, and Desensibilize Nano P) and (2) the evaluation times at two levels (before and after the acid challenge) (*n* = 10). The sample size calculation was based on a medium effect size (*d* = 0.50), with a significance level of 5% and a statistical power of 80%. Representative samples obtained through SEM from each group were used for qualitative analysis of the dentin (*n* = 2).

### 2.2. Selection of Teeth and Acquisition of Dentin Blocks

The research project was submitted and approved by the Ethics Committee on Animal Use of the School of Dentistry of Araçatuba, São Paulo State University (FOA—UNESP #2019-646). Fifty bovine incisors were selected from animals aged between 24 and 36 months. After extraction, the teeth were mechanically cleaned using periodontal curettes (Quinelato, Rio Claro, Brazil) and then subjected to prophylaxis with pumice stone and water. To prevent bacterial proliferation, the clean teeth were stored in a physiological saline solution containing 0.1% thymol and stored at approximately 4 °C until the start of the study.

After cleaning, the crowns were separated from the roots at the cementoenamel junction using a precision cutter Isomet 1000 (Buehler, IL, USA) under water cooling. Subsequently, the crowns were again subjected to the cutter to obtain dentin blocks measuring 4 mm in width by 4 mm in length. Both surfaces were smoothed by manual polishing with #400 and #600 abrasive papers (T469-SF-Noton, Saint-Gobam Abrasives Ltda., Jundiaí, SP, Brazil) until they reached a thickness of approximately 1 mm, measured with a digital caliper (model 500-144B, Mitutoyo Sul América Ltda., Jundiaí, SP, Brazil).

### 2.3. Initial Analysis of Dentinal Fluid Flow/Hydraulic Conductance

All dentin blocks were subjected to a standardization process of initial dentinal fluid flow/hydraulic conductance. First, the specimens were immersed for five min in a 17% EDTA solution (pH 7.4) (Apothicário, Araçatuba, SP, Brazil) to open the dentinal tubules; they were then washed with distilled water and stored in a humid environment until the time of analysis. This procedure is commonly used to maximize dentin permeability. Subsequently, initial dentinal fluid flow and hydraulic conductance measurements were performed to standardize fluid movement through the dentinal tubules. For the analysis, each specimen was connected to a fluid infiltration system (THDO3d Device, Series WMD 3001, Odeme, Luzerna, SC, Brazil) in order to simulate fluid passage through the dentin under intrapulpal pressure, with a constant working pressure of deionized water at 10 psi. For each analysis, a new air bubble was inserted into the system, and its linear displacement (mm) through the micropipette (100 μL, Eppendorf, Hamburg, Germany) was measured over three min using a digital caliper. This analysis was repeated three times for each specimen. The arithmetic mean of the displacement of the three bubbles in mm was calculated and converted into dentinal fluid flow (Q = μL/min) using the formula Q = (V × D)/(L × T), where V represents the standardized capillary volume (µL), D is the bubble displacement (mm), L is the capillary length (mm), and T is the measurement time (min). Then, the hydraulic conductance (Lp; μL.cm^2^/min.cmH_2_O) was calculated based on the formula Lp = Q/(P × A), where P corresponds to the hydrostatic pressure (cmH_2_O) and A to the exposed dentin surface area (cm^2^). Hydraulic conductance considers the area of the specimen through which the water passed (area = 0.16 cm^2^), the pressure in the system (10 psi), and the flow volume (100 μL).

Teeth presenting outlier baseline dentinal fluid flow and hydraulic conductance values were excluded to reduce variability and standardize the samples. The blocks whose dentinal fluid flow and hydraulic conductance values remained close to the average were randomly assigned to the five experimental groups using a randomization procedure performed in Microsoft Excel [39] (n = 10).

### 2.4. Application of Desensitizing Agents

Fifty dentin blocks were then divided into five experimental groups according to the desensitizing agent used. Composition, manufacturers, and application protocols of the materials are described in Table 1. All bioglasses used in this study were mixed with distilled water by adding 0.1 g of bioactive glass powder to 1 mL of water in a 2 mL microtube (Microtubes safe-lock, Eppendorf, São Paulo, Brazil), yielding a 1:10 mixture.

Group 1: Specimens did not receive any product application and were stored in artificial saliva (CaCl_2_.2H_2_O, 0.167 g; KH_2_PO_4_, 0.123g; KCl, 11.2 g; (HOCH_2_)_3_CHN_2_, 2.42 g; distilled water; pH 7.0) (Apothicário, Araçatuba, SP, Brazil) for 15 consecutive days.

Group 2: Specimens received active application of Bioglass^®^ 45S5 (NovaMin^®^ Technology, Alachua, FL, USA) (scrubbing) on the dentin surface using a microbrush applicator (Cavibrush, Joinville, Brazil and remained in contact with the product for 20 min daily for 15 consecutive days.

Group 3: Specimens received active application of Bioglass^®^ F18 (scrubbing) on the dentin surface using a microbrush applicator and remained in contact with the product for 20 min daily for 15 consecutive days.

Group 4: Specimens received active application of the Biosilicate gel (scrubbing) on the dentin surface using a microbrush applicator and remained in contact with the product for 20 min daily for 15 consecutive days.

Specimens received active application of the desensitizing agent Desensibilize Nano P (FGM) (scrubbing) on the dentin surface using a microbrush applicator and remained in contact with the product for 20 min daily for 15 consecutive days.

During the 15 days of treatment, after 20 min of contact with the different desensitizers described above, the specimens from groups 2, 3, 4, and 5 were individually stored in artificial saliva.

### 2.5. Analysis of Dentinal Fluid Flow and Hydraulic Conductance After Treatment with Desensitizers

The analysis of dentinal fluid flow and hydraulic conductance after the application of different desensitizing agents on the dentin was performed as previously described. All evaluations were conducted in a blinded manner.

### 2.6. Citric Acid Challenge

To simulate acidic conditions commonly found in the oral environment, such as demineralization due to erosion, after the treatment under neutral conditions described above with the desensitizing agents, the same dentin specimens were tested for the remineralization effect of the products through dentin permeability analyses before and after the acid challenge.

The dentin blocks were subjected to an acid challenge cycle for seven days following the protocol outlined below: immersion in a 0.5% citric acid solution (1 mL/sample, pH 2.5) (Apothicário, Araçatuba, SP, Brazil) six times a day for two min, under agitation. Between immersions, the dentin blocks were stored in artificial saliva at 37 °C.

### 2.7. Final Analysis of Dentinal Fluid Flow and Hydraulic Conductance

The final analysis of dentinal fluid flow and hydraulic conductance after the application of different materials on the same dentin samples was performed as described previously.

### 2.8. Scanning Electron Microscopy (SEM)

Two specimens per group (*n* = 2) were sputter-coated with gold–palladium (Desk V; Denton Vacuum, Moorestown, NJ, USA) at energies ranging from 3 to 10 keV (40 mA, 60 s) and analyzed using a scanning electron microscope (EVO LS-15, Carl Zeiss, Oberkochen, Germany) to obtain images at 5000× magnification (20 kV) for qualitative dentin analysis.

### 2.9. Statistical Analysis

The dentinal fluid flow and hydraulic conductance data were subjected to the Shapiro–Wilk normality test and showed a normal distribution. They were then analyzed using two-way repeated-measures ANOVA (desensitizing agents and evaluation time), followed by Tukey’s test for multiple comparisons (*α* = 0.05).

## 3. Results

The dentinal fluid flow results are presented in Table 2. The control group showed the highest value in the final readings after the acid challenge compared with the other time points analyzed (*p* = 0.0002). The groups treated with Biosilicate Gel and Desensibilize Nano P showed the highest dentinal fluid flow values after product application compared with the initial and final readings (*p* < 0.0001). There was no statistically significant difference between the evaluated time points for dentin treated with Bioglass^®^ 45S5 and Bioglass^®^ F18 (*p* = 0.0685 and *p* = 0.2549, respectively).

When comparing the groups, after the application of the products, the lowest dentinal fluid flow values were found in the control groups, Bioglass^®^ 45S5, and Bioglass^®^ F18, with statistically significant differences compared to the other groups (*p* < 0.0001). After the acid challenge (final readings), no statistically significant difference was observed between the groups (*p* > 0.05). However, all experimental groups exhibited numerically lower dentinal fluid flow values than the control group at the end of the analysis.

The results of the hydraulic conductance of the dentin are presented in Table 3. The Control and Bioglass^®^ 45S5 groups showed higher values of hydraulic conductance in the final readings after the acid challenge, compared to the readings taken after the application of the treatments (*p* = 0.002 and *p* = 0.0011, respectively). The groups where Biosilicate Gel and Desensibilize Nano P were applied showed the highest hydraulic conductance values after the application of the respective products, compared to the initial and final readings (*p* < 0.0001). There was no statistically significant difference between the time points evaluated for the dentin treated with Bioglass^®^ F18 (*p* = 0.6918).

In the comparison among the analyzed products, after application, the lowest values of hydraulic conductance were found in the control, Bioglass^®^ 45S5, and Bioglass^®^ F18 groups, with a statistically significant difference compared to the other groups (*p* < 0.0001). After the acid challenge (final readings), no statistically significant difference was observed between the groups (*p* = 0.0591). At the end of the analysis, all experimental groups exhibited permeability values that were numerically equal to or lower than those of the control group.

**Figure 1:**
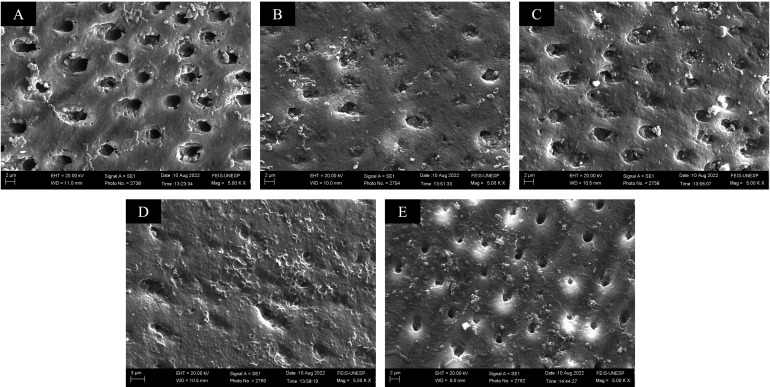
Scanning electron microscopy (SEM) images of dentin surfaces before acid cycling (5000×): (**A**) control (sound dentin); (**B**) Bioglass^®^ 45S5; (**C**) Bioglass^®^ F18; (**D**) Biosilicate Gel; (**E**) Desensibilize Nano P.

**Figure 2:**
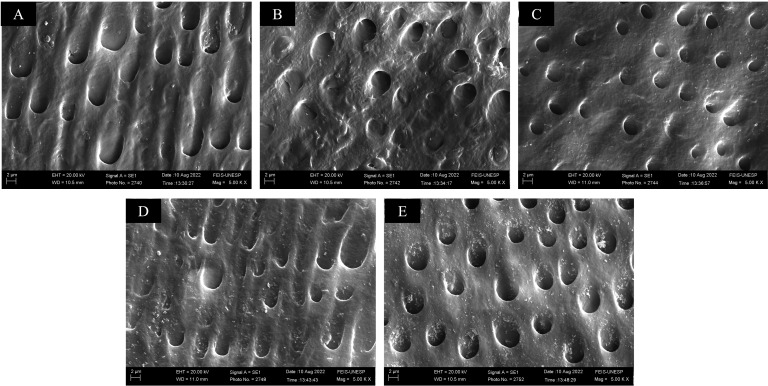
Scanning electron microscopy (SEM) images of dentin surfaces after acid cycling (5000×): (**A**) control (untreated dentin); (**B**) Bioglass^®^ 45S5; (**C**) Bioglass^®^ F18; (**D**) Biosilicate Gel; (**E**) Desensibilize Nano P.

Figure 1 and Figure 2 show scanning electron microscope images of all experimental groups after product application (Figure 1) and after the acid challenge (Figure 2). Qualitatively, the different desensitizing agents appeared to obliterate the dentinal tubules, even after the acid challenge. However, following the acid challenge, the physical structure of the dentin appeared similar among all groups.

## 4. Discussion

Hydraulic conductance and dentinal fluid flow are related but distinct parameters used to evaluate the performance of desensitizing agents. Dentinal fluid flow refers to the movement of fluid through dentinal tubules and is influenced by the degree of tubule openness or occlusion. In contrast, hydraulic conductance is a quantitative parameter calculated from fluid flow under standardized pressure and surface area conditions, representing the ease with which fluid passes through dentin. Therefore, while dentinal fluid flow represents the actual movement of fluid across dentin, hydraulic conductance provides a standardized measure of dentin permeability and is directly associated with dentin hypersensitivity according to the hydrodynamic theory [6].

Considering Brännström’s theory [1,5], which suggests that pain-inducing stimuli increase the flow or alter the direction of dentinal tubular fluid, thereby stimulating nerves around the odontoblasts and causing sensitivity, the dental products proposed to treat this condition aim to suppress nerve impulses by either mechanical or chemical blockage of the dentin tubules or by directly stopping the nociceptive transduction/transmission that occurred within dentin–odontoblast–nerve terminal complex of the dental pulp [40]. In this context, bioactive materials have been studied due to their ability to occlude dentinal tubules after reacting with body fluids, depositing hydroxycarbonate apatite on demineralized collagen fibrils [23,41,42]. This study analyzed four desensitizing agents (one conventional, already marketed, and three experimental bioactive glass solutions), which generally reduced dentin permeability [1] and had their effects altered after acid challenge (Table 2 and Table 3), rejecting both null hypotheses of the study.

Regarding the performance of the desensitizing agents tested, the quantitative analyses conducted in this study (dentinal fluid flow and hydraulic conductance) showed that the lowest values were found for the control, Bioglass^®^ 45S5, and Bioglass^®^ F18 groups, with a statistically significant difference compared to the other groups (*p* < 0.05; Table 2 and Table 3). However, none of the products resulted in a reduction in dentinal fluid flow and hydraulic conductance after treatment (Table 2 and Table 3). This corroborates previous results reported in the literature, suggesting similar efficacy for tubular occlusion among commercially available desensitizing products [43,44,45,46] and experimental bioactive glasses [1,47]. Current understanding of the mechanism of action of bioactive glasses suggests that they promote remineralization through ion release and osteoconductivity, leading to the formation of hydroxycarbonate apatite precipitates that facilitate both mineral deposition and mechanical occlusion of dentinal tubules [20,35,47,48]. However, despite these well-established mechanisms, the specific differences in performance and behavior among the various types of bioactive glasses are still not fully understood.

The results in Figure 1B–E are consistent with this finding, as treated groups exhibited surface coverage of dentinal tubules. The literature has shown that bioactive glasses can lead to occlusion of approximately 90% of dentinal tubules, which may be relevant for the management of hypersensitivity [49]. Before acid cycling, the surfaces appeared rough and particle-rich, suggesting possible deposition of bioactive material over the dentinal surface. Following acid challenge, the surfaces became smoother and more homogeneous (Figure 2B–E), which may indicate partial dissolution or removal of loosely bound particles. Furthermore, the literature shows that, following the discovery of the first bioactive glass and the demonstration of its potential applications, subsequent compositions were developed with modifications primarily focused on production methods and compositional adjustments. Among the materials identified in the present study as exhibiting lower dentinal fluid flow and hydraulic conductance, Bioglass^®^ 45S5 consists of calcium, sodium, phosphate, and bioactive phyllosilicate components, which promote hydroxyapatite formation and support the remineralization of dental hard tissues through interaction with physiological fluids [50]. In contrast, Bioglass^®^ F18 represents a more recent development aimed at expanding the clinical applicability of bioactive glasses, exhibiting improved mechanical properties as well as enhanced angiogenic and anti-inflammatory potential and being classified as highly bioactive [51].

Regarding Biosilicate gel [52], a bioactive glass-ceramic, the material has been proposed as a promising therapeutic agent for enamel and dentin regeneration due to its ability to promote rapid hydroxyapatite deposition on the treated surface. Its particles appear to react quickly with the surrounding environment within the dentinal microchannels, favoring dentinal tubule occlusion [47]. However, despite its recognized bioactive potential, the present findings suggest that Biosilicate Gel may require a longer period for hydroxyapatite maturation and crystallization to achieve effective tubule sealing. In the early stages after application, the ionic dissolution process and the predominantly superficial deposition of mineral precipitates may have limited the immediate reduction in dentinal fluid movement, which could explain the higher dentin permeability and hydraulic conductance values observed after treatment. In the present study, the material showed higher values of dentinal fluid flow and hydraulic conductance after its application, comparing the initial and final readings (*p* < 0.05; Table 2 and Table 3). Similar performance (*p* > 0.05; Table 2 and Table 3) was observed for the Desensibilize Nano P material group, which contains sodium fluoride, nanometric hydroxyapatite, and potassium nitrate in its composition and works through diffusion along the dentinal tubules [53]. The possible role of fluoride and potassium nitrate as active agents in Desensibilize Nano-P should also be considered. Desensibilize Nano P appears to act primarily through neural desensitization mediated by potassium nitrate rather than through immediate dentinal tubule obliteration, which could explain the limited reduction in fluid flow immediately after application. Although nanohydroxyapatite and fluoride may promote remineralization, the mineral deposition formed at the evaluated time may not have been sufficient to produce effective tubule sealing (Figure 1E). It has been discussed that potassium salts are capable of inactivating intradental nerves [53]. However, this principle has not been conclusively demonstrated.

When comparing the evaluation times “post-treatment” and “post-acid challenge,” all the tested desensitizing agents showed some stability/resistance to acid (Table 2 and Table 3). Similar results were reported by Jung et al. (2019) [47] and Garofalo et al. (2019) [54] when evaluating the effects of desensitizing products on dentinal tubule occlusion and erosive wear [47,54]. After the acid challenge, in the final readings, no statistically significant difference was found among the groups (*p* > 0.05). The Biosilicate Gel and Desensibilize Nano P groups not only demonstrated acid stability but also showed a significant reduction in dentinal fluid flow and hydraulic conductance after the challenge (*p* < 0.05). Such a result suggests a possible difference in the effects of the active ingredients and metabolic activity of these products [55].

The present study demonstrated that bioactive glass-based desensitizing agents reduced dentinal fluid flow and hydraulic conductance, suggesting their potential application for the management of dentin hypersensitivity. Among the evaluated materials, Bioglass^®^ 45S5 and Bioglass^®^ F18 showed the most stable performance over time, maintaining reduced permeability and hydraulic conductance values even after the acid challenge. In contrast, Biosilicate Gel and Desensibilize Nano P exhibited higher values after application, suggesting a less stable or less homogeneous tubule occlusion pattern. Although no statistically significant differences were observed among the groups after the erosive challenge, all experimental treatments showed numerically lower dentinal fluid flow and hydraulic conductance values than the untreated control, indicating a protective effect against dentinal fluid movement.

Taken together, these findings suggest that bioactive glass formulations, particularly Bioglass^®^ 45S5 and Bioglass^®^ F18, may promote a more stable occlusion of dentinal tubules and may be useful as potential alternatives for dentin hypersensitivity management under acidic conditions. A limitation of the present study is the use of bovine dentin, which may not fully reproduce the structural characteristics and permeability of human dentin. Differences in dentinal tubule morphology and mineral composition could influence the interaction and deposition behavior of bioactive glasses on the dentin surface. In addition, under the present in vitro experimental conditions, the model does not fully simulate the oral environment, where saliva, mechanical wear, and dietary acids may affect the long-term stability of tubule occlusion promoted by bioactive glass-based desensitizing agents. Therefore, caution is necessary when extrapolating these findings to clinical conditions. Further in situ and clinical studies are recommended to confirm the durability and clinical effectiveness of these materials in the oral environment.

## 5. Conclusions

The evaluated bioactive glass-based desensitizing agents reduced dentinal fluid flow and hydraulic conductance, suggesting dentinal tubule occlusion. Bioglass^®^ 45S5 and Bioglass^®^ F18 showed more stable performance under the conditions of this study, maintaining lower values even after the acid challenge. Although no significant differences were observed among the groups after the erosive challenge, all experimental treatments demonstrated numerically lower values than the control group, suggesting a protective effect against dentinal fluid movement and that they may be relevant for the management of dentin hypersensitivity.

## Figures and Tables

**Table 1:** Composition, manufacturers, and application protocols of the materials.

Group	Composition	Manufacturer	Protocol of Application
*G1—Control* (artificial saliva)	CaCl_2_.2H_2_O, 0.167 g; KH_2_PO_4_, 0.123 g; KCl, 11.2 g; (HOCH_2_)_3_CHN_2_, 2.42 g; distilled water; pH 7	Apothicário, Araçatuba, São Paulo, Brazil	Immersion of samples in 1 mL solution during 15 consecutive days.
*G2—Bioglass^®^ 45S5* (10%)	SiO_2_, CaO, Na_2_O, P_2_O_5_	NovaMin^®^ (GlaxoSmithKline, Alachua, FL, USA)	Active application of the product using a microbrush; product remained on sample for 20 min; removal of excesses with cotton ball; 1˟ a day during 15 consecutive days.
*G3—Bioglass^®^ F18* (10%)	SiO_2_, Na_2_O, K_2_O, MgO, CaO, Au, Ag, B_2_O_3_, P_2_O_5_, ZnO, SrO	Vitrovita, São Carlos, São Paulo, Brazil	
*G4—Biosilicate gel* (10%)	SiO_2_, Na_2_O, CaO, P_2_O_5_	Vitrovita, São Carlos, São Paulo, Brazil	
*G5—Desensibilize Nano P*	Nanometric calcium phosphate (as hydroxyapatite), sodium fluoride (approximately 2%), potassium nitrate 5%	FGM, Joinville, Santa Catarina, Brazil	

**Table 2:** Dentinal fluid flow (Lp; mean ± standard deviation) before and after the application of different desensitizing agents and after acid challenge.

	Initial	After Treatment	Final
*G1—Control*	0.405 ± 0.050 B b	0.393 ± 0.058 B b	0.635 ± 0.217 A a
*G2—Bioglass^®^ 45S5*	0.366 ± 0.055 A b	0.463 ± 0.124 A b	0.447 ± 0.137 A a
*G3—Bioglass^®^ F18*	0.436 ± 0.078 A ab	0.549 ± 0.239 A b	0.450 ± 0.145 A a
*G4—Biosilicate Gel*	0.450 ± 0.049 B ab	0.864 ± 0.180 A a	0.495 ± 0.176 B a
*G5—Desensibilize Nano P*	0.537 ± 0.164 B a	0.840 ± 0.173 A a	0.550 ± 0.156 B a

Means followed by different letters, uppercase in the row and lowercase in the column, show a statistically significant difference between them (*p* < 0.05).

**Table 3:** Hydraulic conductance (Lp; mean ± standard deviation) of dentin before and after the application of different desensitizing agents and after acid challenge.

	Initial	After Treatment	Final
*G1—Control*	0.022 ± 0.003 B b	0.022 ± 0.003 B b	0.035 ± 0.012 A a
*G2—Bioglass^®^ 45S5*	0.020 ± 0.003 B b	0.025 ± 0.007 B b	0.035 ± 0.012 A a
*G3—Bioglass^®^ F18*	0.024 ± 0.004 A ab	0.026 ± 0.007 A b	0.025 ± 0.008 A a
*G4—Biosilicate Gel*	0.025 ± 0.003 B ab	0.047 ± 0.010 A a	0.027 ± 0.010 B a
*G5—Desensibilize Nano P*	0.029 ± 0.009 B a	0.046 ± 0.009 A a	0.030 ± 0.009 B a

Means followed by different letters, uppercase in the row and lowercase in the column, indicate a statistically significant difference between them (*p* < 0.05).

## Data Availability

The raw data supporting the conclusions of this article will be made available by the authors upon request.
